# Metric properties of the “prescribe healthy life” screening questionnaire to detect healthy behaviors: a cross-sectional pilot study

**DOI:** 10.1186/s12889-016-3898-8

**Published:** 2016-12-07

**Authors:** Paola Bully, Alvaro Sanchez, Gonzalo Grandes, Haizea Pombo, Ma Soledad Arietalenizbeaskoa, Veronica Arce, Catalina Martinez, on behalf of the PVS Group

**Affiliations:** Primary Care Research Unit of Bizkaia, Basque Health Service-Osakidetza, Luis Power 18, 4ª planta, E-48014 Bilbao, Spain

**Keywords:** Lifestyle, Mass screening, Primary health care, Risk reduction behavior, Sensitivity and specificity, Validation studies

## Abstract

**Background:**

Feasible and valid assessment of healthy behaviors is the first step for integrating health promotion in routine primary care. Therefore, the aim of this study was to develop and evaluate the validity and reliability of the “prescribe healthy life” screening questionnaire, a brief tool for detecting physical activity levels, consumption of fruit and vegetables, tobacco use and patients’ compliance with minimal recommendations.

**Methods:**

An observational cross-sectional study to determine the reliability and validity of this questionnaire by means of mixed (qualitative and quantitative) methods. Thirteen healthcare professionals designed the questionnaire. One hundred and twenty-six patients from three primary care health centers within Osakidetza (Basque Health Service, Spain) filled in the “Prescribe Healthy Life” Screening Questionnaire and completed an accelerometry record, the PREDIMED Food Frequency Questionnaire and a co-oximetry as gold standards for physical activity, dietary intake and tobacco use, respectively. Correlations, sensitivities, specificities, likelihood ratios and test-retest reliability were calculated. Additionally, the feasibility and utility of the questionnaire were evaluated.

**Results:**

Both reliability and concurrent validity for the consumption of fruit and vegetables (r_spearman_ = 0.59, r_spearman_ = 0.50) and tobacco use (r_spearman_ = 0.76, r = 0.69) as their overall performance in the detection of unhealthy diet (accuracy = 76.8%, LR + = 3.1 and LR- = 0.31) and smokers (accuracy = 86.8%, LR + = 6.1 and LR- = 0.05) were good. Meanwhile, the reproducibility (0.38), the correlation between the minutes of physical activity (0.34) and LR+ (1.00) for detection of physical activity were low. On average the questionnaire was considered by patients easy to understand, easy to fill in, short (5–6 min) and useful.

**Conclusion:**

The “Prescribe Healthy Life” Screening Questionnaire, PVS-SQ, has proved to be a simple and practical tool for use in the actual context of primary care, with guarantees of validity and reliability for the diet and tobacco scales. However, the physical activity scale show unsatisfactory results, and alternative questions ought to be tested.

**Electronic supplementary material:**

The online version of this article (doi:10.1186/s12889-016-3898-8) contains supplementary material, which is available to authorized users.

## Background

Unhealthy behaviors are major contributors to costly chronic health conditions, the main causes of mortality, morbidity, disease burden and overload of healthcare services in industrialized countries [1, 2]. The impact of lifestyle on health is undeniable and effective healthy lifestyle promotion interventions are available. However, this is not a fundamental part of routine primary care practice. The lack of integration is mainly due to two reasons [3]: a) changing people’s habits is not an easy task, since behavior is determined by multiple personal, institutional and environmental factors; b) the difficulty of changing routine clinical practice of professionals and organization of Primary Health Care (PHC) services in a context of overwork and lack of time and training. For the above reasons, interventions on habits and behaviors under actual primary care consultation conditions turn into complex tasks [4, 5].

With the strategic aim of designing, assessing and routinely practicing PHC instruments, techniques and innovative, feasible and effective strategies for tackling and managing the most important healthy behaviors and lifestyles, the Primary Care Research Unit of Bizkaia (UIAPB) has created the research-action project “Prescribe Healthy Life” (PVS in Spanish: “Prescribe Vida Saludable”) [6]. PVS programs are based on socio-cognitive, transtheoretical models of stages of change and intervention strategy for the 5 As (Assess, Advise, Agree, Assist, and Arrange follow-up) [7]. PVS programs have started to work on the three habits that are at the top of causes of disease burden in Spain in 2013, namely, levels of physical activity, diet and tobacco use [8]. The initiative emphasizes the importance and need for standardized measures of these three key behavioral determinants to be recorded in electronic health records (EHRs) because assessing patient-reported risk behaviors is a critical first step of the 5As strategy and is necessary to measure progress.

In spite of the fact that obtaining valid and suitable measurements of physical activity, diet and tobacco use is a complex task, we can find several both objective and subjective procedures in the scientific literature. In the case of physical activity [9] procedures based on behavioral observation, time-motion analysis (accelerometers, pedometers, GPS, mobile applications, miniature video cameras and other tracking devices), physiologic stress response evaluation (heart rate monitor, Doubly Labeled Water [DLW]), time or daily records and several kinds of interviews and questionnaires (either self-administered or undertaken by a trained interviewer) are all noteworthy. To measure the effect of diet, the most commonly used procedures are [10] the Diet Record, 24-h Recall and food frequency questionnaires (FFQ) that ask about the usual frequency of consumption of a group of foodstuffs or nutrients [11, 12]. Biomarkers and technology-based methods for dietary assessment also prove to be useful as additional measures [13, 14]. However, these intensive measures are typically not included given their invasiveness and expense. As for tobacco use, the most commonly used detection procedures in primary care are co-oximetry, determination of cotinine in blood and/or saliva, detection of thiocyanate in blood, saliva or urine and the patient’s statement [15]. To systematically collect these measures successfully it is imperative for them to be standardized, valid, practical, feasible, actionable, applicable to multiple groups and consistent with these broader practice redesign initiatives. These characteristics can only be provided by relying on a brief and self-reported measure whose aim is the determination of compliance with minimal recommendations for healthy habits.

There were several screening instruments for healthy habits in UK and US primary care contexts [16]. However, some tests were developed for research purposes, others were too long, there was no single instrument or procedure that was optimal for all risk factors or populations even though multiple risk factor screening is currently feasible and available (e.g. Case-finding and Help Assessment Tool (CHAT) [17], My Own Health Report (MOHR) [18], etc.) and none of the tests was validated in our population. In an attempt to resolve these problems and obtain representative data on compliance with minimal recommendations for healthy habits in the Basque autonomous community, our research team developed a multidimensional instrument to detect physical activity levels, healthy diet and tobacco use in the second stage of the PVS program, the “Prescribe Healthy Life” Screening Questionnaire (PVS-SQ) (Cuestionario de Cribaje “Prescribe Vida Saludable”, in its original version).

The aim of this study was to develop and evaluate the validity and reliability of the results obtained with this instrument in the Spanish population, in order to assist in the diagnosis and therapeutic decision-making within the PVS intervention program.

## Methods

### Design

Transversal observational study on the validation and determination of the reliability of the results obtained with a brief instrument for detection of healthy habits, framed within the PVS quasi-experimental clinical trial. The study was performed according to the STAndards for the Reporting of Diagnostic accuracy studies (STARD) statement for diagnostic tests [19].

### Sample

Two-stage sampling was carried out to attain the number of necessary participants. During an initial phase, a daily sample of 18 patients aged 10 to 65 years old, stratified by age and sex, from the total of patients attending the collaborating PVS centers, was selected by the Basque Public Health Service data center using systematic sampling. In a second phase, those who consented to participate as indicated in the telephone PVS study measurement interview, belonging to three of the eight collaborating healthcare centers, were included in this substudy.

Individuals were excluded if they regularly participated in sports such as swimming or cycling, activities that cannot be measured by the accelerometer, or if they proved unwilling to take part in the study. Patients were also excluded if they had a cardiovascular disease; musculoskeletal problems that could be exacerbated by exercise; any major chronic respiratory, renal or liver disease; an infectious or metabolically unstable condition; cognitive problems; severe emotional distress; complicated pregnancy; or reasons for being unlikely to comply with follow-up procedures.

### Instruments

The list of instruments used in the validation study can be split into three differentiated blocks:

“Prescribe Healthy Life” Screening Questionnaire (PVS-SQ)

This is a questionnaire, designed, implemented and evaluated in Spanish, comprising 12 multiple choice items of which 6 measure physical activity, 2 measure consumption of fruit and vegetables, 1 is about tobacco use and 3 refer to other aspects. It can be completed in different ways (phone survey, on paper, on the Osakidetza website and in mobile applications); all of them are connected to the patient’s clinical history (Osabide-AP) (See Additional file 1). In order to allow to non Spanish speakers to understand the questionnaire, we created an English-language copy. The PVS-SQ items were subjected to a back-translation process in order to achieve linguistic equivalence. Two independent professional translators translated each item of the Spanish version into English. Subsequently, the item translations were compared and discussed to arrive at a single version. Next, two different translators translated the English version into Spanish. After comparison of the two versions, a single Spanish-language version was obtained. Experts in assessment compared each item of the original Spanish version with the inversely-translated version, explored the possible lack of meaning equivalence between the two, and made some changes to the English copy. See Additional file 2.

The correction of the PVS-SQ questions is done in two phases:

The first phase serves to estimate levels of weekly physical activity, the number of servings of fruits and vegetables consumed daily and the status regarding the use of tobacco.

A) In questions related to physical activity, the number of days of light, moderate or vigorous physical activity is multiplied by the central value of the daily minutes declared. For example if the patient has declared five days of moderate physical activity for 20 to 29 min a day, we consider he/she does about 125 (5 × 25) minutes of moderate PA a week. B) In the diet questions, first the values 0, 1, 2, 3, 4 and 5 are assigned to the response categories “not every day,” “1 serving per day,” etc., respectively. The sum of the servings of vegetables and pieces of fruit consumed per day is written below. C) For the tobacco question, tobacco consumption is rated by assigning the value 0 to the answers “non-smoker” and “former smoker,” 1 to “occasional smoker,” 2 to “trying to quit smoking” and 3 to “smoker.”

The second phase in the correction of PVS-SQ serves to know if patients meet the minimum recommendations in the 3 habits assessed. The criteria used for compliance were:

A) The minimum public health PA recommendations, that is, at least 30 min of moderate PA 5 days per week, or at least 20 min of vigorous intensity PA 3 days per week [20]. In the PVS-SQ questionnaire, this corresponds to having marked the combinations formed by the number of days of moderate PA as 5, 6 or 7 and the number of minutes each day 30–39 or 40 or more and/or having marked the number of days of vigorous PA between 3 and 7 and the number of minutes per day between 20–29 and 40 or more. B) Consume 5 or more portions of fruit and/or vegetables a day. It is met if the sum of the values of the two diet questions gives a value of 5 or higher. C) Complete abstention from tobacco, i.e., having marked non-smoker or former smoker options for at least one year.

#### Reference measurements

##### Physical activity (PA)

To measure physical activity objectively, 15 ActiGraph GT3X (ActiGraph, Pensacola, FL, USA) accelerometers were used [21]. The GT3X monitor is lightweight (27 g), compact (3.8 cm × 3.7 cm × 1.8 cm) and rechargeable (i.e. lithium polymer battery-powered) [22]. It must be worn at the waist using a belt clip or elastic belt. It uses a solid-state tri-axial accelerometer to collect motion data on three axes, i.e., vertical (Y), horizontal right–left (X) and horizontal front–back (Z) axis. The ActiGraph also includes the vector summed value ([*x* ^2^ + y^2^ + z^2^]^(1/2)^), known as “vector magnitude.” The GT3X measures and records time-varying accelerations in the range ∼ 0.05–2.5 Gs. Accelerometer output is digitized by a twelve-bit analog to digital converter (ADC) at a rate of 30 Hz. Once digitized, the signal passes through a digital filter that band-limits the accelerometer to the frequency range 0.25–2.5Hz. Each sample is summed over an “epoch,” that is, a specific time interval which typically corresponds to 60 s except in younger populations, in which shorter epochs are recommended [23]. The ActiGraph output is given in “counts,” with one count equaling 16.6 milliGs^s−1^ at 0.75 Hz. Activity counts, which are the result of summing the absolute values of the sampled change in acceleration measured during the time period (dA/dL), represent a quantitative measure of activity over time. The counts obtained in a given time period are linearly related to the intensity of the subject’s PA during this period and can be translated into minutes of moderate to vigorous physical activity using cut-offs of 1952 and 5725 allowed, respectively [24]. One hour of record will not be considered valid if the number of consecutive minutes with 0 “counts” is greater than 30 min. Data from accelerometers were considered valid if the monitor was used at least 4 of the 7 days and for at least 10 h each day [25, 26]. The reference standard used to confirm if patients were physically active or not also was the minimum public health PA recommendation [19].

##### Fruit and vegetables (F&V)

Dietary intake was assessed with a semi-quantitative PREDIMED food frequency questionnaire (FFQ) with 136 items validated in our setting [27–29]. For each food item, a commonly used portion size was specified (slice, glass, teaspoon, etc.), and participants were asked how often they had consumed that unit on average over the previous year. Emphasis was added to ensure that answers were related to long-term dietary exposure and not to recent changes in diet. Nine options for frequency of consumption ranging from no intake to more than six times a day were offered. The selected frequency item was converted to a daily intake. For example, if a response was 5–6 times a week, it was converted to 0.78 servings per day (5.5 week/7 days). Healthy consumption was considered 5 or more portions of fruit and/or vegetables a day and compliance with at least 9 of the 14 recommendations for a Mediterranean diet.

##### Tobacco (TB)

Tobacco use was objectively measured using co-oximetry. Co-oximetry is a clinical test to detect the loss of hemoglobin oxygenation capacity and it consists of determining the level of carbon monoxide or CO in the air exhaled by an individual [15]. The co-oximeter (Micro IV Smokerlyzer brand, Bedfont Scientific, Rochester, UK) was used for this; this is a high precision monitor to measure CO concentration in ppm (parts per million). It has a concentration range of 0–250 ppm of CO and resolution +/−2 ppm. The cut-off point is 6 ppm of CO; lower or equal and higher values are categorized as non-smoker and smoker, respectively.

#### Feasibility and utility

The feasibility and utility of the PVS-SQ for patients were evaluated by the following four questions, which could be rated on a scale of 1 to 10: 1) Was the questionnaire easy to understand?; 2) Was the questionnaire easy to fill in?; 3) Was the questionnaire long? and 4) Do you find the results of the questionnaire useful?

### Procedure

Evidence that guarantees the inferences constructed from the test was sought over two stages. Qualitative and quantitative techniques were used in phases 1 and 2, respectively.

During the first phase, content validity and face validity were supported by expert judgment and based on Anglo-Saxon instruments which have proved to be more useful, valid and reliable [16, 30]. A multidisciplinary panel of subject matter experts was convened to complete this phase (see PVS group for a complete list of panel members in Additional file 2). For each of the three behaviors, small working groups from the multidisciplinary panel examined available measurement tools. The working groups were instructed to consider a set of scientific and practical criteria in making their recommendations: 1) among the scientific criteria, reliability and evidence of validity, applicability (intercultural studies; validation in different languages) and sensitivity to change in the tests were considered; 2) among the practical considerations, the premise was the feasibility of its generalized use in the context of primary care (shortness, ease of use for patients and the staff and low cost).

Analysis by each one of the clinical committees led to a pool of possible items for each habit studied. These items were reviewed by means of an iterative process and as a result 12 received approvals from the group of experts. The scale related to the minimum recommended level of physical activity was prepared considering the results obtained in the framework of the Multicenter Clinical Trial Experimental Program for Promotion of Physical Activity in PHC (PEPAF in Spanish) [31]. This scale was applied by family doctors in routine primary care consultation and revealed a positive predictive value of 87.6% for the detection of insufficiently active patients. In regard to diet, the procedure used was the formulation of two succinct questions focused on the frequency of daily ingestion of fruit and vegetables given that they are the foodstuffs which revealed the greatest discriminatory power and the most commonly used reporting procedure [16, 30]. Fruit and vegetable intake is also an indicator of a healthy overall diet. Specifically, total fruit (whole fruit and 100% fruit juice) and whole fruit intake are the second and third most correlated factors with an overall healthy eating pattern, respectively, after amount of empty calories consumed [32]. Finally, tobacco use was identified by means of classifying the patient into the following self-declared categories: non-smoker, former smoker, smoker, occasional smoker or trying to quit smoking. This questionnaire, PVS-SQ, was initially administered by doctors, nurses and patient care in the healthcare centers during the stage to assess habits from the PVS program.

**Fig. 1:**
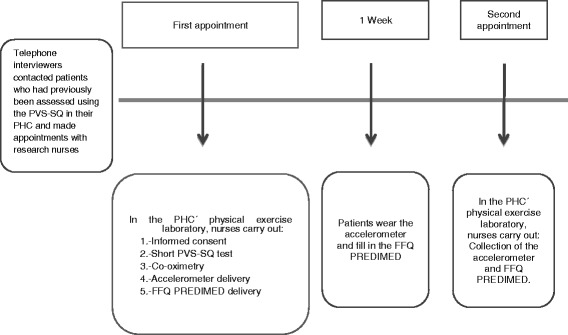
Timeline of measurement and data collection

During phase 2, which lasted from June to December 2013, three research nurses were trained on arrangement of visits, implementation of the test, use of accelerometers and co-oximeters, and data download. Data quality was assured throughout the study by the Primary Care Research Unit of Bizkaia. The timeline for study procedures is shown in Fig. 1. First, professional interviewers contacted patients who had previously been assessed using the PVS-SQ in their PHC by telephone and two separate appointments were made. The first appointment was within 15–30 days. In both appointments, the patient met with the research nurse in person. The patient gave written informed consent, completed the PVS-SQ again, underwent the co-oximetry test and height and weight were taken as inputs for the GT3X accelerometer. Nurses instructed patients on how to wear the accelerometer correctly and gave them a copy of the PREDIMED FFQ to fill in at home. During the following 7 days, the patient wore the accelerometer continuously, except in the shower. At the second appointment, accelerometer data were downloaded to the computer by the research nurse and the patient delivered the full PREDIMED FFQ. At this visit, the patient was asked about the period of time when the accelerometer had been worn. Additionally, the feasibility and utility of the questionnaire were evaluated in an incidental subsample of 33 patients.

If the person does not meet the recommendations (A1: Assess), the health professional provides personalized advice (A2: Advice) on the benefits of these habits, the risks associated with their absence and recommendations to be achieved. Next, targets are jointly agreed with the patient (A3: Agree). If the patient agrees and is prescribed a behavior modification plan (A4: Assist), monitoring is programmed and continues for one year (A5: Arrange follow-up).

### Analysis

The reliability or accuracy of the measurement was quantified in terms of reproducibility. The reproducibility or ability of the test to offer the same results when its application is repeated under similar circumstances was determined by means of the Spearman correlation. The quantitative study of validity included: first, the calculation of concurrent validity, that is, the association of scores from the PVS-SQ scales with scores obtained for the reference measurements. Based on prior studies, a correlation higher than 0.40 was considered good. Second, the diagnostic efficiency indices to detect unhealthy habits: sensitivity (Se), specificity (Sp), accuracy, positive likelihood ratio (LR+) and negative likelihood ratio (LR-). Analyses were performed in all cases to verify whether the metric characteristics of the scales were affected by the sex of patients or the healthcare center of origin. The feasibility and utility of the questionnaire were evaluated using frequency analysis and indices of central tendency and dispersion. SAS (v. 9.2, SAS Institute, Cary, NC, USA) was used to perform all statistical analyses.

## Results

### Participants

In this study, 781 patients were invited to take part; 589 declined to participate and 192 agreed to participate, of whom only 126 (16.17%) attended the appointment. The characteristics of the 126 patients included in the analyses are shown in Table 1. There were 63 (50%) women whose mean age was 44.11 years (SD = 12.37) and 63 (50%) men, mean age 38.44 (SD = 13.56); 42 (31.75%), 44 (34.92%) and 40 (33.33%) belonged to health centers A, B and C, respectively. According to the accelerometer, 22.2% of patients met the minimum physical activity recommendations; in accordance with the diet questionnaire only 4.2% had a healthy diet and according to the co-oximeter, 80.9% did not smoke. There are two people for whom their clinical characteristics are unknown.

**Table 1:** Sociodemographic and clinical characteristics of patients

	Number	Percent
Age		
10–19 years	7	5.56
20–29 years	20	15.87
30–39 years	29	23.02
40–49 years	38	30.16
50–59 years	19	15.08
60–65 years	13	10.33
Level of studies		
no studies	2	1.59
Primary studies	18	14.29
Elementary post-secondary education, secondary school graduate, basic general education up to year 8	24	19.05
Secondary education, professional training, advanced secondary education	22	17.46
2nd Degree Professional training, Advanced Level Training Cycle	19	15.08
Diplomas	7	5.56
Graduate degrees, higher technicians	34	26.98
Main situation		
Active worker	65	51.59
Unemployed having worked previously	16	12.70
Seeking first job	5	3.97
Retired, pensioner or independently wealthy	6	4.76
Studying	15	11.90
Houseworker	10	7.94
Disabled	7	5.56
Other situations	2	1.59
Cancer		
No	124	100.00
Cardiovascular Pathologies		
No	110	88.71
Yes	14	11.29
Mental Disorders		
No	113	91.13
Yes	11	8.87
Musculoskeletal Pathologies		
No	116	93.55
Yes	8	6.45
Respiratory Pathologies		
No	123	99.19
Yes	1	0.81
Gastrointestinal Pathologies		
No	122	98.39
Yes	2	1.61
Neurologic Pathologies		
No	117	94.35
Yes	7	5.65
Metabolic Pathologies		
No	111	89.52
Yes	13	10.48
Other Pathologies		
No	114	91.94
Yes	10	8.06
Comorbidity		
No pathologies	81	65.32
1 pathology	25	20.16
2 pathologies	10	8.06
3 pathologies	7	5.65
4 or more pathologies	1	0.81

### Reliability

A total of 71.42% (*n* = 90) of patients filled in the PVS-SQ both times, with an average time gap between them of 122.74 days (DT = 202.14). The degree of association between both measurements was moderate to low, moderate to high, and high in regard to weekly minutes of moderate to vigorous physical activity, number of portions of fruit and vegetables consumed each day, and tobacco use categories, respectively (Table 2).

**Table 2:** Test-retest reliability of PVS screening

	Time 1 Mean (*SD)*	Time 2 Mean (*SD)*	Spearman correlation r (n; p)
PA	135.31 (127.79)	125.62 (108.15)	0.38 (*n* = 73; *P* < 0.001)
F&V	3.21 (1.99)	2.92 (1.88)	0.59 (*n* = 77; *P* < 0.001)
TB	0.70 (0.45)	0.68 (0.46)	0.76 (*n* = 90; *P* < 0.001)

### Validity

#### Concurrent validity

##### Physical activity

For the total sample, the minutes of at least moderate physical activity recorded by the PVS-SQ (M = 124.59) revealed a moderate-high correlation (r[120] = 0.34; *P* < 0.001) with the minutes of similar activity recorded by the accelerometer (M = 141.50). The analyses revealed a statistically significant effect of the interaction between predictive power of the PVS-QS PA scale and the healthcare center of origin (F[2,114] = 6.77; *P* = 0.002). The degree of association was low, moderate-low and moderate-high for healthcare center A, B and C users, respectively.

##### Fruit and vegetables

For the sample as a whole the number of fruit and vegetable portions declared in the PVS-SQ (M = 2.92) and those recorded in the PREDIMED FFQ (M = 1.40) revealed a moderate correlation (r[107] = 0.54; *P* < 0.001). The interaction between sex and number of portions measured by the F&V scale of the PVS-QS was statistically significant (F[1,103] = 4.72; *P* = 0.032); the degree of linear association between answers given by women and men was moderate-high and low, respectively.

##### Tobacco

Moderate-high and high correlations were estimated between the category of tobacco use self-declared in the PVS-SQ TB scale and the CO level (in ppm) in exhaled air measured by co-oximetry (r[121] = 0.69; *P* > 0.001). Interactions with sex and center were not statistically significant (Table 3).

**Table 3:** PVS-SQ Concurrent validity

	PA			F&V			TB		
	PVS-SQ M (SD)	Accelerometer M (SD)	r (n)	PVS-SQ M (SD)	FFQ PREDIMED M (SD)	r_Spearman_ (n)	PVS-SQ M (SD)	Co-oximeter M (SD)	r(n)
Sex									
Men	137.15 (104.73)	156.09 (160.62)	.30* (*n* = 57)	2.39 (1.89)	1.20 (1.21)	.27* (*n* = 55)	0.63 (0.49)	0.76 (0.43)	.64* (*n* = 59)
Women	110.95 (110.62)	127.35 (136.29)	.36* (*n* = 63)	3.43 (1.74)	1.63 (1.45)	.65* (*n* = 52)	0.72 (0.45)	0.81 (0.40)	.70* (*n* = 62)
HC									
A	103.50 (107.07)	145.53(135.71)	.14* (*n* = 39)	2.55 (1.64)	1.39 (1.29)	.49* (*n* = 31)	0.52 (0.50)	0.77 (0.42)	.57* (*n* = 40)
B	152.38 (110.20)	84.11 (98.14)	.39* (*n* = 42)	3.07 (2.12)	1.32 (1.38)	.48* (*n* = 39)	0.78 (0.41)	0.83 (0.38)	.86* (*n* = 42)
C	116.50 (103.95)	200.95 (182.29)	.63* (*n* = 39)	3.15 (1.80)	1.49 (1.49)	.70* (*n* = 37)	0.71 (0.46)	0.76 (0.43)	.74* (*n* = 38)
Total	124.59 (108.32)	141.50 (148.86)	.34* (*n* = 120)	2.92 (1.88)	1.40 (1.38)	.50* (*n* = 107)	0.67 (0.47)	0.79 (0.40)	.69* (*n* = 121)

*P < 0.05

### Diagnostic efficiency index

Table 4 reveals, from left to right, the values of confounding matrices, in which the screening result is shown against the condition of patients measured by the reference tests and the diagnostic indices obtained to detect physical inactivity, insufficient consumption of fruit and vegetables and smoking measured by the PVS-SQ.

**Table 4:** PVS-SQ Diagnostic Efficiency Index

	TP	FP	FN	TN	Se, % (95% CI)	Sp, % (95% CI)	Accuracy, % (95% CI)	LR+ (95% CI)	LR- (95% CI)
PA	74	21	15	5	81.3 (72.1–88.0)	19.2 (8.5–37.8)	67.5 (58.6–75.3)	1.0 (0.8–1.3)	0.88 (0.5–1.6)
F&V	90	1	27	3	76.9 (68.5–83.6)	75.0 (30.0–95.4)	76.9 (68.6–83.53)	3.1 (0.6–16.8)	0.31 (0.2–0.4)
TB	24	15	1	81	96.0 (80.5–99.3)	84.4 (75.6–90.3)	86.8 (79.6–91.7)	6.1 (3.8–9.8)	0.05 (0.0–0.3)

*TP* true positive, *F* false positive, *FN* false negative, *TN* true negative, *NE* not estimable

The sensitivity or likelihood of correctly classifying people with unhealthy habits was high for the 3 scales; 81.3%, 76.9% and 96% for physical inactivity, insufficient consumption of fruit and vegetables and smoking, respectively. Specificity was low for physical activity (19.2%), average for consumption of fruit and vegetables (75%) and high for abstinence from smoking (84.4%). This led to accuracy values that range from 67.5 to 86.8%. Likelihood ratio incorporates both sensitivity and specificity and is a direct estimate of how much the test result changes the odds of having the condition. All 6 questions designed to detect physical inactivity had low LR+ and LR-. For the questions regarding insufficient consumption of fruit and vegetables, both LR+ and LR− showed limited discriminating power. The smoking issue had LR+ > 5, which indicates that it is a good test for “ruling in” the condition and LR− < 0.1 which indicates that it is a very good test for “ruling it out.”

**Fig. 2:**
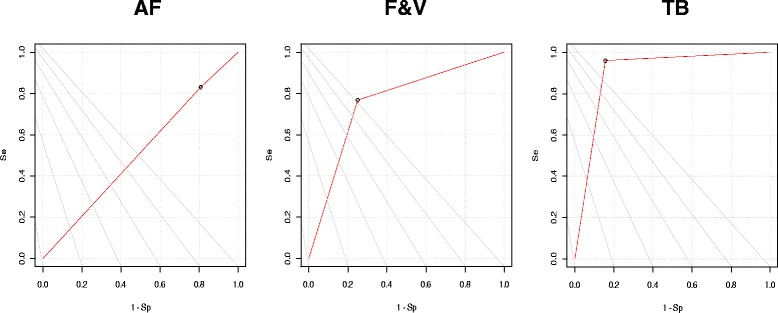
PVS-SQ ROC curves

Graphically, Fig. 2 shows the Receiver Operating Characteristic (ROC) curves that reveal the relationship between sensitivity (y-axis) and specificity (x-axis) for each one of the PVS-SQ scales.

### Feasibility and utility

**Fig. 3:**
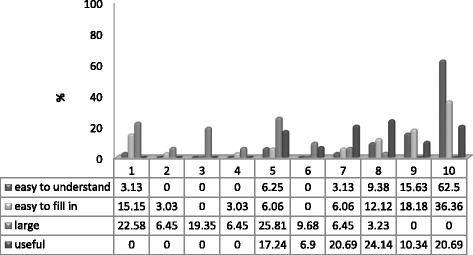
Feasibility and utility scores distribution

Usually patients completed the instrument in 5–6 min and the internal dropout range for each of the 12 questions was lower than 5%. Patients and health care providers responded positively to the screening tool. The distribution of scores on the four questions that assess the feasibility and utility of PVS-SQ perceived by the patients is shown in Fig. 3.

On average it was considered easy to understand (M = 8.97; SD = 1.99) and complete (M = 7.30; SD = 3.30). The greatest difficulties occurred because the computer application worked through the internet and sometimes the connection was cut off before completion of the test. When asked about the duration, on average, it was considered short (M = 3.77; SD = 2.08). Most importantly, the results obtained with the test were perceived as quite or very useful (M = 7.65; SD = 1.71). Participating providers stated that the PVS-SQ was helpful for facilitation and planning of the visit, as well as for guiding further questioning.

## Discussion

Currently, there is no doubt over the effectiveness of interventions for the promotion of healthy habits and their priority in the healthcare system given the reduction in chronic diseases and, consequently, costs and increased quality of life gained in the medium to long term. For the promotion of health under routine primary care conditions to become reality, the first step is being able to detect those people who comply with/breach the most important healthy lifestyle habits. The PVS-SQ was created with this aim; it has proved to be a simple, practical measurement instrument with minimal guarantees for it to be used in the context of primary care systems.

More specifically, in regard to the scale for measuring physical activity, analyses have reported: moderate to low reproducibility; moderate concurrent validity with statistically significant differences according to healthcare center of origin, with a lower degree of association in the healthcare center whose population had a lower deprivation index and a higher immigration rate (center A); suitable sensitivity for detection of sedentary lifestyle but low specificity and LR+. This limits the usefulness of the physical activity scale with discriminatory purposes. The results obtained with the sample as a whole are in accordance with those found in other studies that tested the validity, within the range of correlations coefficients 0.21–0.85, and test-retest reliability, ranged between 0.17 and 0.99, for self-report measures to assess physical activity [17, 33–36]. It is possible that higher reliability coefficients would have been encountered had the time elapsed between test and retest been shorter.

For the consumption of fruit and vegetables scale all psychometric indicators -reproducibility, concurrent validity, sensitivity, specificity, LR+, LR- and area under the curve ROC- revealed moderate values. The degree of association between the number of portions of fruit and vegetables consumed measured by the PVS-SQ and PREDIMED FFQ presented statistically significant differences according to sex, with a moderate to high association for women and low association for men. Reliability is similar to that found in other studies made outside the context of PHC, ranging between 0.52 and 0.95 [37, 38]. Other studies confirmed the pattern of men consuming fewer servings of fruit and vegetables daily than women [37, 39–41], and found similar differences (2.52 vs. 3.47; *P* < 0.01) in Baker et al. [40]. Sex differences in concurrent validity are in line with what was found in other studies [37, 42]. However, correlations found in our study are higher and statistically significant for both men and women in accordance with those found by Serdula et al. [42]. Finally, two similar questions have high specificity and identify more than 80% of individuals with biomarker profiles indicative of low fruit and vegetable intake [43].

For tobacco use, the results revealed excellent performance both in regard to reproducibility and concurrent validity and diagnostic efficiency indices without any statistically significant differences according to sex or healthcare center of origin. These results are similar to those found by Barrueco et al. [15] and indicate that the scale can be considered a correct measure that accurately reflects tobacco consumption in the general population in our setting.

### Study limitations and strengths

#### Limitations

The first involves the instrument: a) the fruit and vegetable consumption scale captures only two aspects of nutrition but is highly correlated with health outcomes. In fact, consumption of low levels of fruits and vegetables (less than 400 grams per day) is considered to be among the top 10 risk factors for global mortality, resulting in 1.7 million global deaths annually [44]; b) The tobacco measure is also limited in that it only asks questions about cigarette use and does not ask about tobacco exposure (e.g., whether the patient lives with someone that smokes indoors) and c) the questions about motivation to change lifestyle habits and belly size were not included in the validation study because it was considered that they were not directly related to the screening of the 3 behavioral habits of healthy life with which the PVS programs began working.

Second, because this was a cross-sectional study, it was not possible to test screeners’ sensitivity to change in healthy habits over time, an important issue in behavioral intervention studies.

Third, it is possible that the long period between test and retest (on average 123 days) evaluates not only the screening instrument but also natural behavioral changes.

Finally, our selection process was not a systematic patient-recruiting method, so there might be some bias in the data. However, validity and reliability studies typically use convenience samples and sample size in the current study was larger than in many other ones.

#### Strengths

The measure includes a lack of clarity regarding the term “portion” in F&V consumption. Results from the Bensley et al. study [45] suggest there are differences in responses to frequency questions on fruit and vegetable intake depending on whether information about serving size or portion is provided. Occasional or intermittent smokers are not missed with these screening questions.

The PVS-SQ is very quick to fill in, can be used in different settings (healthcare centers, companies, schools, etc.). It can be administered in several ways by different professionals (doctors, nursing personnel, teachers, etc.) or be self-administered. In all cases results are recorded in the patient’s EHR which facilitates subsequent management of the healthy habit promotion by the healthcare professional.

### PVS-SQ usefulness

Physical activity, diet and tobacco use are modifiable determinants of health. If individual healthcare providers have information on their patients’ lifestyle patterns, they can recommend that their patients use ancillary services such as lifestyle counseling and they can motivate and advise them to increase their physical activity, eat more fruits and vegetables and reduce tobacco consumption. If the healthcare system has information on its populations’ negative lifestyle patterns, population-level approaches can be used to tackle these patterns. Such approaches have proved successful in several settings, such as schools and communities. If researchers have quality information on lifestyle patterns as part of the EHR, studies could identify the most effective interventions that should be used in primary care clinical practice [46] and evaluate related costs and potential differential effects across patient populations.

## Conclusions

The “Prescribe Healthy Life” Screening Questionnaire, PVS-SQ, has proved to be a simple and practical tool for use in the actual context of primary care, with guarantees of validity and reliability, but whose dimension of physical activity could be improved.

This study highlights a critical gap in the area of physical activity assessment claiming that there is still a lack of short and easy-to-administer physical activity measures with a robust validity and reliability associated with their use. The best options so far are accelerometers and other procedures based on time-motion analysis and physiological markers. However, because time and burden continue to be important factors for many researchers and practitioners, there is still a continued need to modify and/or develop new physical activity screeners and assess them for validity and reliability.

## Additional files

Additional file 1: Prescribe Healthy Life Screening Questionnaire. (DOCX 556 kb)
Additional file 2: Prescribe Healthy Life Screening Questionnaire English Copy. (DOCX 916 kb)
Additional file 3: PVS Group Members. (DOCX 37 kb)

## Abbreviations

DLW: Doubly labeled water
EHRs: Electronic health records
F&V: Fruits and vegetables
FFQ: Food frequency questionnaire
FN: False negative
FP: False positive
HC: Health center
LR-: Negative likelihood ratio
LR+: Positive likelihood ratio
NE: Not estimable
PA: Physical activity
PHC: Primary health care
PVS: Prescribe healthy life, in Spanish
PVS-SQ: “Prescribe healthy life” screening questionnaire
Se: Sensibility
Sp: Specificity
TB: Tobacco
TN: True negative
TP: True positive
