# Supplementary figures and images for: Metric properties of the “prescribe healthy life” screening questionnaire to detect healthy behaviors: a cross-sectional pilot study
Source: BMC Public Health. 2016 Dec 7;16:1228. doi: 10.1186/s12889-016-3898-8 (PMC5142282; doi:10.1186/s12889-016-3898-8)

**Additional file 1**


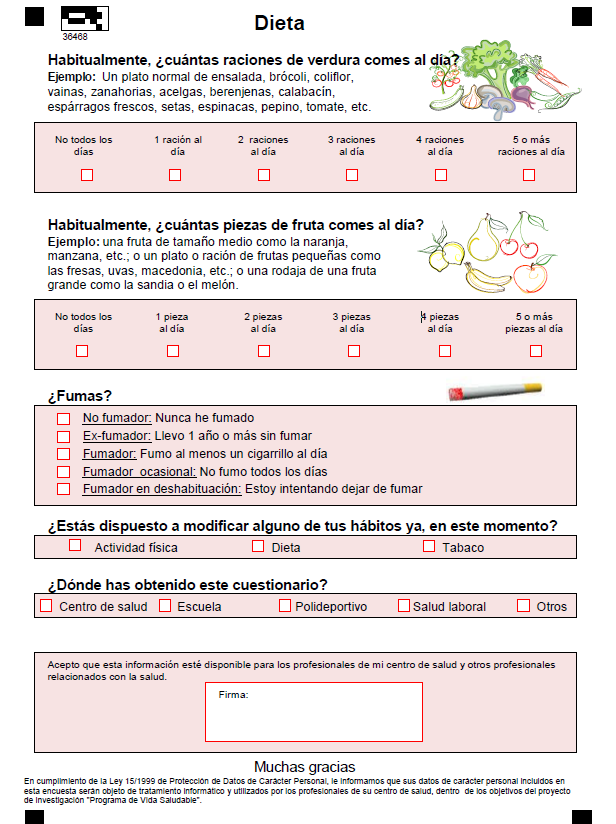

Supplement: Additional file 1: — Prescribe Healthy Life Screening Questionnaire. (DOCX 556 kb) [file 12889_2016_3898_MOESM1_ESM.docx]
