# Supplementary material for: Metric properties of the “prescribe healthy life” screening questionnaire to detect healthy behaviors: a cross-sectional pilot study
Source: BMC Public Health. 2016 Dec 7;16:1228. doi: 10.1186/s12889-016-3898-8 (PMC5142282; doi:10.1186/s12889-016-3898-8)
Supplement: Additional file 3: — PVS Group Members. (DOCX 37 kb) [file 12889_2016_3898_MOESM3_ESM.docx]

**Additional file 3**

**PVS Group Members:**

Leading Team:

*Primary Care Research Unit of Bizkaia, Basque Health Service–Osakidetza* (principal investigator: Gonzalo Grandes; co-investigators: Alvaro Sánchez, Haizea Pombo, Josep Maria Cortada, Catalina Martinez, Paola Bully, Aitor Sanz-Guinea).

Basque Health Service–Osakidetza:

*Deputy Director of Healthcare Services* (Carlos Sola),

*Department of Information Technology* (Martín Begoña, Susana Iglesias, Maite Cuadrado, Nuria Gonzalez)

*Management of Goierri- Alto Urola District* (Director: Teresa Garmendia; Mª Luz Jáuregui; Amaia Hernando),

*Beasain Health Center* (Coordinator: Justo Múgica; Maria Pilar Alberdi, Mª Ángeles Arrondo, Amaia Azkoitia, Xabier Epaizabal, Mª Aranzazu Echeverria, Mª Esperanza García, Mª Ángeles García, María Erkuden Imaz, Mª Antonia Iparraguirre, Mª Isabel Irizar, Mª Rosario Larrea, Mª Dolores López, Petra Pacheco, María Yolanda Porres, Begoña San Juan, Mª Aranzazu Suquia, Mª Teresa Arrospide, Carolina Díez, Miren Arantxa Igartua, Oihana Jauregui, Alazne Saizar, Mª Jose Tilves, Mª Lourdes Etxeberria, María Aurora Valdivielso, Xabier Mugica, Mª Mercedes Lasagar, Coro Zabaleta),

*Management of Bilbao District* (Director: Jesús Larrañaga, Maribel Romo, Pilar Isla),

*La Merced Health Center* (Coordinator: Mª Isabel Urcelay, Mary Helen Corrales; Mª Ángeles Crespo, Javier José María Jesús De Ordozgoiti, María Iciar Elguezabal, Susana Esteban, Catalina Frau, Laura Gallo, Inés Yolanda Martín, Nerea Ordorika, José Ramón Pérez, Mª Begoña Relloso, María Soledad Sangroniz, María Iluminada Santos, Patxi Xabier Iturbe)

*Management of Interior District* (Director: Enrique Maíz, Cristina Domingo, Carmen Esparta)

*Matiena Health Center* (Coordinator: Esther Gorostiza; Mª Esther Azpitarte, Bixente Barrutia, Amaia Bengoa, Francisco José Miguel, Ana Isabel Etxebarria, Mª Belén García, Mª Jose Ibars, Mª Jose Lasa, Mª Carmen Martínez, Maura Pernudo, Lourdes Oribe, Mª Dolores Ustarroz, Valentina Camino, Leire Corpión, Leire Ortuondo, Mª Carmen López, Rosana Abraldes, Eneko Ibarruri, Javi Alonso)

*Management of Uribe District* (Director: Mª Luz Marqués; Encarnación San Emeterio, Anton Elorriaga),

*Sondika Health Center* (Coordinator: Enrique de la Peña; Mª Carmen Artola, Teresa Casado, Jesús García, Mª Paz Sánchez, Luisa Santos, María Lanzarote)

Basque Government Health Department: (Concha Castells; Francisco Cirarda, Henar Ortuondo, Pilar Manrique, Ines Urieta, Amaia Ajuria)

Clinical Committees:

*Physical Activity*: Ricardo Ortega, Jesus Torcal, Mª Soledad Arietaleanizbeaskoa, Verónica Arce, Alvaro Sánchez, Gonzalo Grandes

*Diet*: Bittor Rodriguez, Pilar Amiano, Esther Gorostiza, Enrique de la Peña, Alvaro Sánchez, Gonzalo Grandes

*Smoking cessation*: Esther Azpitarte, Mary H. Corrales, Josep Cortada, Alvaro Sánchez, Gonzalo Grandes

Community:

*Beasain:*

Arcelormittal company (Juan Manuel Elosegui)

CAF company (Iñaki Korta, Ainhoa Irastorza, Leire Makibar)

Antzizar sports center (Jon Alkaiaga, Karmele Alkaiaga)

*La Merced:*

Community Health of Bilbao City Council (Iñaki Aldamiz)

School Health of Bilbao City Council (Virginia Zelaia)

Miribilla Primary Education Center (Adela Etxeberria, Itziar Basurto)

Bilbao sports center (Gonzalo Casado, Maite Martínez, Alberto Díez)

Municipal Office of Bilbao La Vieja, San Francisco y Zabala (Javier Rojo)

“Bakuba” Community association (Sara Garteiz)

“Etorkinekin Bat” Community association (Aitziber Artabe, Ainhoa Parra)

“Iniciativa gitana” Community association (Marcelo Borja, Mª Carmen Jiménez)

*Sondika:*

Sondika Council (Gorka Carro, Bernardo Valdivielso)

Municipality of Sondika (Janire Kasuso, Esther Martin)

Gorondagane Primary Education Center (Paulino Parra)

Txorierri Secondary Education Institute (Loinaz Albizu)

Txorierri polytechnic (Marivi Cuartango)

Sondika sports center (Unai Atxa)

Olarra company (Jesús Miguel Enríquez)

Sondika Pharmacy (Rosario Acebal, Javier Ancel)

*Matiena:*

Matiena Council (José Luis Navarro, Inmaculada Zapardiez)

Commonwealth municipality of Abadiño ( Nerea Lejarzaburu)

Traña Matiena Primary Education Center (Edurne Madariaga)

Abadiño Secondary Education Institute (Eugenia Peral)

Abadiño sports center (Pablo Mas)

Mutualia company (Juan Mayor)

FREMAP company (Joseph Reverte)

Estampaciones metálicas company (Bernard Mandaluniz)

Others:

Basque Institute for Healthcare Innovation: O+berri (Roberto Nuño)

Osarean (Josu Llano)

Osatek SA (Enrique Gutiérrez)

University of Colorado, School of Medicine, Department of Family Medicine (Maribel Cifuentes)
